# Mr. Davie's Cases of Phthisis, Cured by Uva Ursi

**Published:** 1806-04

**Authors:** L. Davie

**Affiliations:** Framlingham, Suffolk


### *To the Editors of the Medical and Physical Journal.*

Gentlemen,

I Beg leave to request your insertion of the following facts, which, as they relate to the cure of a disease that has invariably resisted the use of the best directed means, I think it of importance to my professional brethren, and mankind in general, and that it is my duty to bring it forward, unequal as I may feel to the task of doing it *properly*.

Mrs. P——, a widow Lady, at Laxfield, between the age of 30 and 40, had been for some time labouring under symptoms of Phthisis; she was attended by myself and Mr. Hinchman, an elderly and very eminent surgeon in this neighbourhood; we both considered her case as consumption, arising from the suppuration of tubercles, and a variety of means were had recourse to without effect. The disease went rapidly on; the expectoration was purulent, and very copious; the pulse in the evening at 120, and never lower than a 100; the hectic was regularly formed, and diarrhœa alternated with morning sweats; the lancinating pains of the thorax, with which she had been long distressed, disappeared about this time.—In this state she was visited by Dr. Hamilton, an eminent and scienitfic physician, at Halesworth, who considered her case as one not within the power of medicine, but advised a trial of the Uva Ursi. It was begun with in doses of a ℈j. ter die, in a weak infusion of the angustura, and increased to a ʒß. ter die. From this time an amendment began to take place; her pulse became less frequent; her cough and expectoration lessened; in fine, all appearances of disease, after a month's perseverance in the Uva Ursi, were either removed, or so much abated, that she considered herself well, and has continued so since last November.

Another case has more recently occurred to me of a similar kind, but of a more active nature, and more rapid in its progress; the disease was as clearly marked, and has been as happily relieved; the expectoration here, was considerably streaked with blood.

Such is a faithful account of the success of Uva Ursi in these cases. I leave it for those who possess more ability than myself, to explain how a medicine seemingly possessing so little power, compared with many others we are in the habit of prescribing, should act in curing a disease, so formidable in its nature, and in general so fatal in its termination.

I am, &c.

L. DAVIE.

*Framlingham, Suffolk, March* 15, 1806.

